# Label-free multiplex electrochemical immunosensor for early diagnosis of lysosomal storage disorders

**DOI:** 10.1038/s41598-022-13259-1

**Published:** 2022-06-04

**Authors:** Haya Abdulkarim, Mohamed Siaj

**Affiliations:** Department of Chemistry, Université du Québec à Montréal, Montreal, QC H3C 3P8 Canada

**Keywords:** Sensors, Medical and clinical diagnostics

## Abstract

Pompe, Gaucher and Krabbe disease are lysosomal storage disorders (LSDs) which are a group of genetic diseases that causes the accumulation of lipids in tissues and cells. Pompe, Gaucher and Krabbe are characterized by the deficiency of acid α-glucosidase (GAA), β-Glucocerebrosidase (GBA) and galactocerebrosidase (GALC), and treatable if detected in their early stages. Here, we present the fabrication of an electrochemical immunosensor for the multiplexed quantification and simultaneous detection of GAA, GBA and GALC. The sensor was developed by electrodepositing gold nanoparticles (AuNPs) on an array of carbon electrodes, followed by the immobilization of GAA, GBA and GALC specific antibodies via functionalization with cysteamine and glutaraldehyde. The multiplexed immunosensor was able to successfully detect GAA, GBA and GALC at the femtomolar level with respective low detection limits of 0.12 pg/ml, 0.31 pg/ml and 0.18 pg/ml. The immunosensor showed good selectivity, sensitivity and good recovery when spiked in human serum, which confirms its possible applicability in point-of-care testing for the early diagnosis of LSDs.

## Introduction

Lysosomal storage disorders (LSDs) are a group of inherited genetic diseases that causes lipids to accumulate in tissues and cells^1^, resulting from mutations in genes encoding intralysosomal enzymes^2–4^. All LSDs share the same characteristic that they cause accumulation of naturally degraded substrates in lysosomes^5^, they cause the accumulation of substrates leading to destruction and disfunction of cells, consecutively, causing tissue disfunction^2,6^. The severity of the LSDs depends on the nature and quantity of the accumulated substrate^6^. LSD patients appear normal at birth, but they develop symptoms early in childhood^7^. Neurological symptoms include brainstem disfunction and seizures, and peripheral symptoms include kidney and heart injuries, muscle atrophy, ophthalmic diseases, enlargement of liver and spleen and irregular bone development^8^. However, treatment is available for most LSDs if discovered early in the infantile stage, such as enzyme replacement therapy (ERT), bone marrow or stem cell transplantation and gene therapy, therefore early detection of LSDs is crucial^6,7^.

Pompe disease (glycogen storage disease type II) is an LSD caused by the deficiency of the lysosomal enzyme acid α-glucosidase (GAA). GAA is necessary in degrading glycogen to glucose thus its deficiency leads to the accumulation of glycogen in organelles^9,10^. As treatment for pompe, acid α-glucosidase enzyme is given to patients as ERT, it breaks down glycogen to glucose to reduce its buildup in cells, however, treatment with acid α-glucosidase should be started as early as possible in infants^11^. Another LSD is Krabbe disease (KD) or Globoid cell leukodystrophy (GCL), is a neurological condition caused by the deficiency of galactocerebrosidase (GALC). GALC enzyme is essential for the degradation of galactosylceramide in the white matter of the cerebrospinal nervous system. Krabbe becomes clinically apparent within 6 months of birth and ends in death within 24 months if not treated^12,13^. Gaucher disease is the most common sphingolipidosis, it results from the deficiency in β-Glucocerebrosidase (GBA)^14^, it causes the accumulation of glucosylceramide in macrophages^15^, and it is classified into three main subtypes based on the presence or absence of neurological involvement^16^. Among all LSDs, Pompe, Gaucher and Krabbe diseases are the most common and have severe symptoms that end with mortality. However, these diseases have treatments available, and are detectable in their infantile stage. Hence, if detected and diagnosed early in the patient’s infantile stage, treatments can be administered accordingly.

It was proven that the initial identification of LSD patients can be achievable by immunoquantification of lysosomal enzymes and proteins, since mutations lead to not only deficiency in the enzyme activity but also cause diminishment in the amount of protein^7^. Deficient patients have protein levels lower than the cut-off concentration which is about 3–100 ng/ml in healthy individuals^17^. Previously used methods for the quantification of some LSD related proteins were reported such as fluorescence, tandem mass spectrometry (LC–MS/MS) and immunoassays like enzyme linked immunosorbent assay (ELISA)^2,7^. Nonetheless, these methods are known to be time consuming and take long analysis time, costly, require specialized laboratories and require large sample volume, making them not ideal for point-of-care testing (POCT)^18^. POCT allows rapid access to results therefore providing faster monitoring, choice of treatment, prognosis and diagnosis of diseases, resulting in better decision making which is often vital to patient’s health. To achieve POCT in the most effective way, methods that are more cost-effective and rapid are being developed^19^.

In order to expedite and facilitate clinical diagnosis and POCT, multiple analysis of different biomarkers produce faster and more accurate results. Thus, multiplexed analysis utilizing a single analytical device holds a great promise in upgrading and simplifying diagnostic procedures, as it provides more data, quicker. Multiplexed detection provides numerous advantages such as utilizing less sample volume, less averaged analysis time, more statistically reliable conclusions and more informative detection outcomes^20^.

Biosensors are evolving to be an interesting cheaper, simpler and more sensitive alternatives for conventional methods of detection for diagnostic purposes. More specifically, electrochemical immunosensors are being researched extensively in the field of biomedical research and diagnostics, due to their high sensitivity, rapid response, minimization of sample volume used, their capability of being miniaturized and their ability of multiplexing. Electrodepositing electrochemical immunosensors with gold nanoparticles (AuNPs) via chronoamperometry, improves its performance via the enhancement of electron transfer rate and catalytic activity of the sensor^21^. It is done by reducing HAuCl_4_ using potassium nitrate. AuNPs increase the surface area allowing more antibodies to immobilize to the surface of the transducer resulting in a significantly higher signal and sensitivity. Gold nanoparticle-modified electrodes demonstrated a nearly threefold increase in electroactive area, resulting in an increase in functional density of biomolecules as well as improved electron exchange and sensitivity^22–24^.

In this work, we report a novel multiplexed electrochemical immunosensor developed for the quantification and simultaneous detection of GAA, GBA and GALC. Carbon microarray disposable chips electrodeposited with AuNPs were utilized due to their high conductivity and high surface area that allows the immobilization of more antibodies. Antibodies for GAA, GBA and GALC were immobilized on the sensor for the detection of the proteins. This multiplexed sensor could be utilized in the crucial early diagnosis of LSDs in newborns in order to administer the right treatment.

## Results and discussion

### Construction of the immunosensing platform

**Figure 1:**
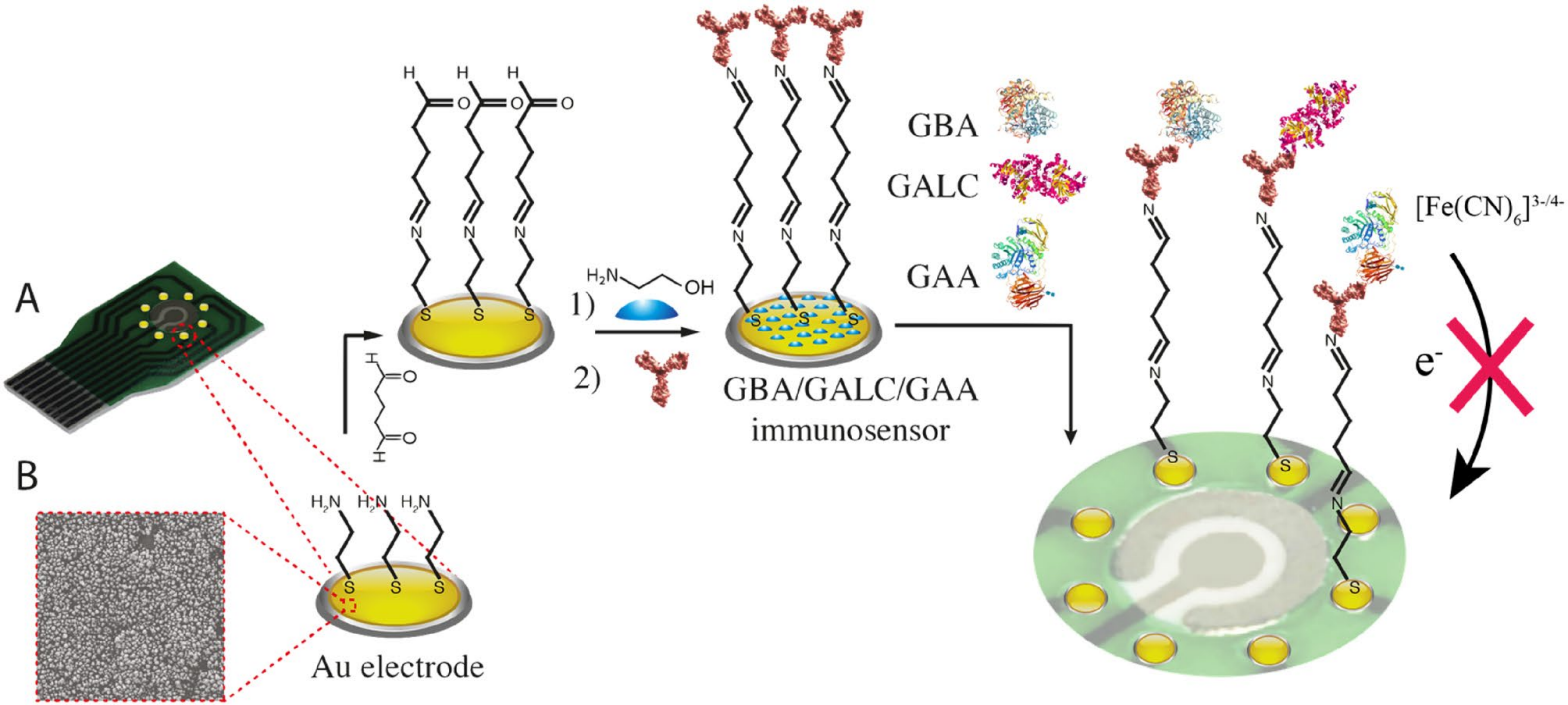
Schematic diagram of the fabrication steps and detection mechanism of the multiplexed immunosensor for GAA, GBA and GALC (**A**). SEM image of an electrode after electrodeposition of AuNPs via chronoamperometry (**B**). The AuNPs-modified electrodes were then functionalized with cysteamine to form SAMs, followed by glutaraldehyde as crosslinker. The binding of the to the antibodies hinders the access of the solution-based redox probe to the electrode surface (red cross).

One-use array carbon electrodes were used to fabricate the multiplexed immunosensor (Fig. 1). The first step was to modify the carbon working electrodes by electrodeposition of Au particles by reducing KNO_3_ solution of HAuCl_4_ by chronoamperometry. When the HAuCl_4_ is reduced, it forms an intense layer of AuNPs on each carbon electrode surface that can be seen clearly. Thiol-gold chemistry was utilized for surface coating of AuNPs, it forms a strong and stable gold-sulfur bond that allows strong anchoring of biological elements. Therefore, AuNPs-modified electrodes were then functionalized with cysteamine to form SAMs, followed by glutaraldehyde which is a homobifunctional crosslinker that binds the amine group from the cysteamine to one of aldehyde groups to form an imine bond, and the other aldehyde group to bind with an amine group from the antibodies. GAA, GBA and GALC antibodies are therefore immobilized on each electrode individually. The immunosensors were then ready to be used for detection after the electrodes were blocked with ethanolamine to eradicate non-specific binding and to prevent false signals. The stability of the immunosensor was examined by preparing an immunosensor and measuring it’s SWV peaks, and then storing it in a humid container at 4 °C for 1 month. After a month, SWV peaks were measured again and the response change before and after storing was found to be only (2.2–3%), which indicates the stability of the immunosensor under long-term storage conditions.

### Electrochemical characterization of the fabrication steps

**Figure 2:**
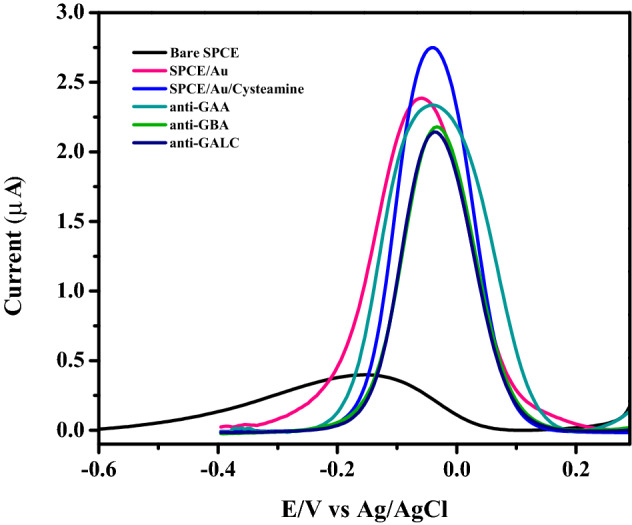
SWV peaks for the characterization of the fabrication steps of the immunosensor before, after AuNP electrodeposition and after immobilization of antibodies and blocking with ethanolamine.

SWV was measured for each step of the immunosensor fabrication, in redox couple solution of Fe(CN)_6_^−3/−4^ as shown in Fig. 2. Bare carbon was measured before electrodeposition with AuNPs, it showed a small peak current. After AuNP deposition a significant increase in current was observed which is attributed to the higher surface area of AuNPs and faster electron transfer compared to carbon. After functionalization with cysteamine and the formation of SAM on AuNPs, there was a further increase in the peak current due to the positive charge of amine groups formed. Following the addition of the crosslinker glutaraldehyde and the immobilization of antibodies for GAA, GBA and GALC, peak currents for each sensor decreased differently because of the conformational and size changes between antibodies.

### Binding of the immunosensor and time optimization of immunocomplex formation

**Figure 3:**
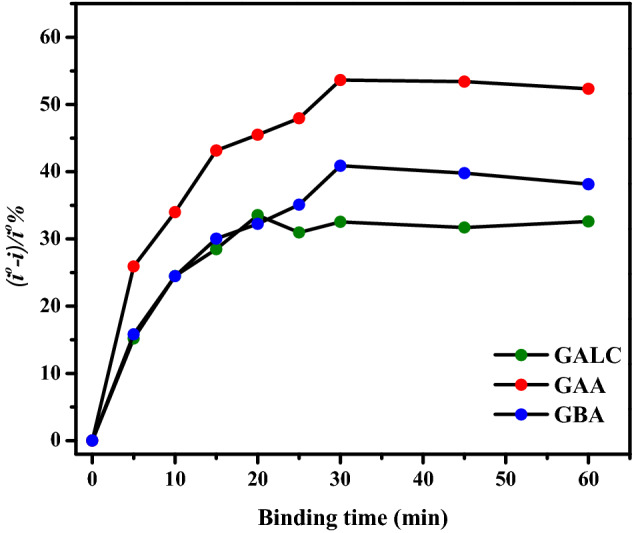
Plot of the immunosensor response ((i° − i)/i° %) versus the binding time in minutes of the immunosensors with their specific proteins for GAA, GBA and GALC.

To optimize the minimal time required for the formation of the immunocomplex to get the maximum signal, GAA, GBA and GALC multiplexed immunosensor was incubated with protein solutions for different time periods starting with 5 min up to 1 h. As shown in Fig. 3, after each incubation, SWV was measured in reference to the signal of the immunosensor. The sensor response was calculated using ((i° − i)/i° %) and a continuous increase in antibody-antigen binding was detected after each incubation. this experiment was repeated three times. After 30 min of incubation no signal enrichment was observed, consequently, the optimum time to form the immunocomplexes were 30 min for GAA and GBA, and 20 min for GALC.

### Immunosensor’s detection of GAA, GBA and GALC

**Figure 4:**
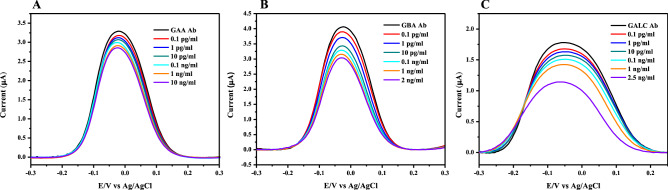
SWV peaks measured before and after binding with different concentrations of GAA (**A**), GBA (**B**) and GALC (**C**).

**Figure 5:**
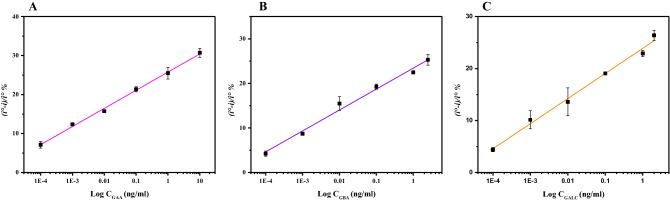
Calibration curves plotting the Log of the different concentrations (ng/ml) of GAA (**A**), GBA (**B**) and GALC (**C**) versus the immunosensor’s response ((i° − i)/i° %). Error bars show the standard deviation of the measurements.

The multiplexed immunosensor was incubated in prepared solutions of different concentrations of the proteins GAA, GBA and GALC. SWV was measured after each incubation to determine the binding of proteins. Figure 4 shows SWV peaks obtained after binding, as the concentration was increasing, the commensurate peaks were decreasing gradually. Figure 5 shows the corresponding calibration curves displaying adequate linearity. GAA showed a linear behavior between 0.1 pg/ml and 10 ng/ml, linear behavior was also observed between 0.1 pg/ml to 2 ng/ml for GBA, and between 0.1 pg/ml to 2.5 ng/ml for GALC. The error bars in calibration curves represent SD of triplicate measurements. For each immunosensor, limit of detection (LOD) was calculated, 0.12 pg/ml (1.5 fM) for GAA, 0.31 pg/ml (5.19 fM) for GBA, and 0.18 pg/ml (2.25 fM) for GALC, indicating the high sensitivity of the immunosensor at the femtomolar level. The LOD values were compared to the detection limits of the commercial ELISA kits available for the proteins, as LODs obtained were 0.9, 12.8 and 78 pg/ml for GAA, GBA and GALC, respectively. The multiplexed immunosensor showed to be superior to ELISA by having a lower LOD, being label-free, faster, cost-effective, ability to simultaneously detect multiple analytes in one run and their promising applicability in point-of-care testing.

### Selectivity of multiplexed immunosensor

**Figure 6:**
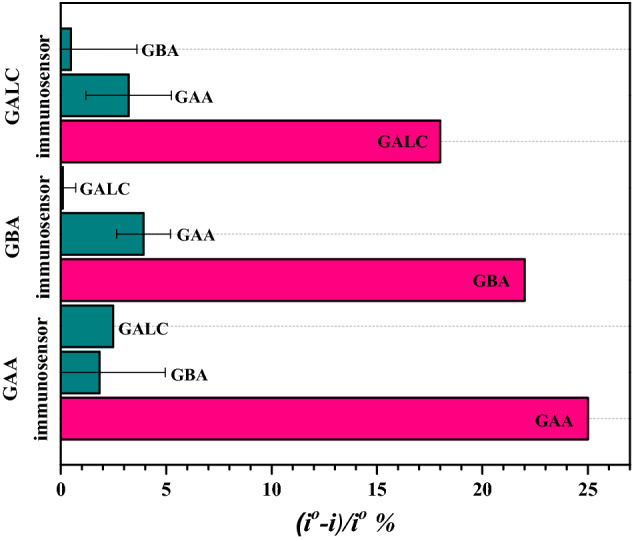
Immunosensor’s responses to 0.5 ng/ml to GAA, GBA, GALC and BSA. Error bars represent SD of the measurements.

**Figure 7:**
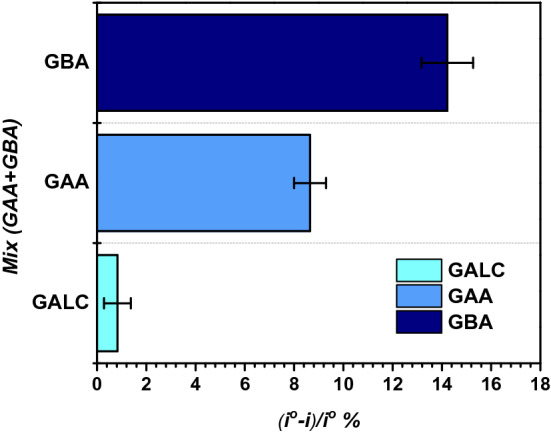
GAA, GBA and GALC sensors’ response after incubation each with a mixture of 0.5 ng/ml of GAA and GBA.

The multiplexed immunosensor’s selectivity was tested by incubating the electrodes in 0.5 ng/ml of GAA, GBA and GALC individually. Each immunosensor response was measured before and after incubation with the non-specific proteins, then washed with PBS pH 7.4. Figure 6 shows that each immunosensor had the highest signal and affinity to their corresponding protein in comparison to the non-significant signal obtained after incubation with the other non-specific proteins. This confirms the high selectivity and specificity and low cross-reactivity of the multiplexed immunosensor against GAA, GBA and GALC. The simultaneous detection was validated by preparing a mixture containing 0.5 ng/ml of GAA and GBA and incubating it on each of the sensors on the chip. Specific significant signals were obtained only for GAA and GBA compared to GALC as shown in Fig. 7, confirming its ability of simultaneous detection.

### Application of the immunosensor in spiked serum

To assess whether the immunosensor would be applicable to test biological samples obtained from patients for rapid and sensitive quantification of GAA, GBA and GALC for the initial diagnosis of LSDs, immunosensors were tested on spiked samples of human serum. Spiked serum samples were also analyzed with commercial ELISA kits (LSBio) specific for GAA, GBA, and GALC for comparison. After measuring each serum sample, recovery was studied by spiking serum samples with GAA, GBA and GALC, the recovery results are shown in Table 1. The good recovery percentages suggest the immunosensor’s high precision and eradicates the significant matrix effect, therefore it’s applicability in different biological specimens.

**Table 1:** Three human serum samples individually diluted 1:100, spiked with 10 ng/ml of GAA and 1 ng/ml of GBA and GALC, and the calculated recovery % and relative standard deviation (RSD %).

	Spiked concentration (ng/ml)	Measured concentration (ng/ml)	Recovery %	RSD %
GAA	10	10.52	105.2	2
GBA	1	0.905	90.5	10.6
GALC	1	0.96	96	1.6

## Experimental

### Materials and reagents

Cysteamine hydrochloride, ethanol amine, potassium ferrocyanide (K_4_Fe(CN)_6_) potassium ferricyanide (K_3_Fe(CN)_6_), glutaraldehyde, phosphate buffered saline tablets (PBS 0.01 M phosphate buffer, 0.0027 M potassium chloride and 0.137 M sodium chloride, at pH 7.4), potassium nitrate, gold chloride solution (HAuCl4), and Human Serum (from male AB clotted whole blood) were obtained from Sigma-Aldrich (Oakville, ON, Canada). ELISA kits for Acid α-glucosidase (GAA), β-glucocerebrosidase (GBA) and Galactocerebrosidase (GALC) were purchased from LSBio (Seattle, WA, US). Proteins and monoclonal antibodies for Acid α-glucosidase (GAA), β-glucocerebrosidase (GBA) and Galactocerebrosidase (GALC) were also acquired from LSBio.

### Instrumentation

All electrochemical measurements were done in 5 mM Fe(CN)_6_^−3/−4^ redox couple prepared in 10 mM PBS buffer solution (pH 7.4). Electrochemical measurements were performed using AUTOLAB pgSTAT302N (Metrohm, Netherlands) potentiostat, connected to a computer with Nova 1.9 software. Multiplexed immunosensors were fabricated using carbon microarray disposable chips, chips and their corresponding connectors were obtained from BioDevice Technology Ltd. (Nomi, Japan). Each array chip contains eight individual working electrodes, a silver/silver chloride reference electrode and a carbon counter electrode. Parameters for square wave voltammetry SWV measurements are (initial potential 0.4 V, end potential − 0.4 V, step potential − 0.005 V, amplitude 0.02 V, frequency 25 Hz, interval time 0.04 s and scan rate 0.125 mV/s.

## Methods

### Au electrodeposition and fabrication of the immunosensor

A disposable array chip of eight working electrodes (BioDevice Technology Ltd., Japan) was electrodeposited with AuNPs using chronoamperometry. A solution of 0.1 M KNO_3_ containing 6 mM HAuCl_4_ was placed on the sensor’s surface, and it was connected to a potentiostat. Chronoamperometry was applied at a potential of − 0.85 V over a period of 200 s for each working electrode until the surface turns visibly golden. Chips were then washed with deionized water and dried then stored for further use.

The multiplexed immunosensor was fabricated on the electrodeposited array chip as indicated in (Fig. 1). First, the array chip containing eight electrodes electrodeposited with AuNP was incubated with 10 mM cysteamine hydrochloride solution for 2 h to form self-assembled monolayers (SAM), then washed with deionized water. Consequently, the modified electrodes were incubated with 2.5% glutaraldehyde solution in phosphate buffered saline (PBS) pH 7.4 for 1.5 h to activate of the surface, then rinsed with PBS pH 7.4. Electrodes were then incubated with 10 µg/ml of GAA, GBA and GALC antibody solutions prepared in PBS pH 8.5, each solution was added to a different working electrode on the same chip, they were kept for 1.5 h. After careful washing with PBS pH 7.4 to remove non-bound antibodies, 0.1 M ethanolamine was added and incubated for 30 min to block any non-reactive sites. After blocking, the chip was rinsed again with PBS pH 7.4 and stored in a water-saturated container at 4 °C. The electrodes were incubated in all steps at room temperature and in a humid environment.

### Response and detection of the immunosensor

Each modified electrode was incubated with different concentrations of their specific proteins (0.1 pg/ml to 10 ng/ml for GAA), (0.1 pg/ml to 2 ng/ml for GBA) and (0.1 pg/ml to 2.5 ng/ml for GALC) in PBS pH 7.4. The electrodes were then washed with PBS pH 7.4 and then measured in redox system of Fe(CN)_6_^−3/−4^ using SWV. For selectivity experiments, electrodes were incubated with 0.5 ng/ml of their other non-specific proteins, followed by measuring the signal. To calculate the sensor’s signal, the percentage of decline in the SWV peaks obtained before and after protein binding to the antibodies was utilized and calculated as ((i° − i)/i° %), where (i) indicates the current after incubation with the proteins, and (i°) is the current measured before incubation at the control concentration of zero.

### Application of GAA, GBA and GALC immunosensors in human serum

Human serum (from male AB clotted whole blood, Sigma Aldrich, Canada) was diluted 1:100 with PBS pH 7.4, divided and spiked with 10 ng/ml of GAA, and 1 ng/ml of GBA and GALC, each was incubated on the chip for 30 min at room temperature and then washed with PBS pH 7.4. SWV was then measured for each spiked sample and recovery % was obtained. Results were compared with commercial ELISA kits for each protein.

### ELISA for GAA, GBA and GALC in serum

Triplicates of ELISA were performed for GAA, GBA and GALC in accordance with the manufacturer’s protocol. Primarily, the standards and spiked diluted serum samples were incubated in the antibody precoated wells at 37 °C for 1 h (GAA and GBA) and 2 h (GALC). After washing, 100 μl of Biotin-conjugated secondary antibodies were incubated in the wells for 1 h at 37 °C. Wells were washed and then incubated with 100 μl of streptavidin–Horseradish Peroxidase (HRP) complex at 37 °C for 1 h (GALC) and 30 min (GAA-GBA). Next, 100 μl of 3,3′,5,5′-Tetramethyl-benzidine (TMB) substrate solution were added to the wells and incubated for 10–20 min, then stopped by adding 50 μl of sulfuric acid stop solution. Readings were taken at 450 nm using a microplate reader.

## Conclusion

In conclusion, a novel multiplexed label-free electrochemical immunosensor was successfully developed for the quantification and simultaneous detection of acid α-glucosidase (GAA), β-Glucocerebrosidase (GBA) and galactocerebrosidase (GALC) for the early detection of LSD diseases; Pompe, Krabbe and Gaucher. This rapid multiplexed sensor was fabricated on AuNPs-modified carbon printed array electrodes. It showed higher sensitivity and lower LOD than commercial ELISA kits. The immunosensor showed very good specificity for each analyte against the other, and a very low LOD of 0.12 pg/ml (1.5 fM) for GAA, 0.31 pg/ml (5.19 fM) for GBA, and 0.18 pg/ml (2.25 fM) for GALC, indicating the high sensitivity of the immunosensor at the femtomolar level, respectively. Very good recovery percentages when measured in spiked human serum, which confirms its possible applicability in POCT for the early diagnosis of LSDs.

## Data Availability

The data that support the findings of this study are available from Pr Siaj (siaj.mohamed@uqam.ca) but restrictions apply to the availability of these data, which were used under license for the current study, and so are not publicly available. Data are however available from the authors upon reasonable request and with permission of Pr Siaj to any researcher wishing to use them for non-commercial purposes. Researchers who wish to obtain a copy of the data submit their request to siaj.mohamed@uqam.ca.
